# Response to Carter et al.

**DOI:** 10.1093/jncics/pkaa016

**Published:** 2020-03-05

**Authors:** Martin Eklund, Kristine Broglio, Christina Yau, Jason T Connor, Allison Stover Fiscalini, Laura J Esserman,

**Affiliations:** p1 Department of Medical Epidemiology and Biostatistics, Karolinska Intitutet, Nobels väg 12, 17177 Stockholm, Sweden; p2 Berry Consultants LLC, 3345 Bee Caves Rd, Suite 201, Austin, TX 78746, USA; p3 Department of Surgery, University of California San Francisco, 1600 Divisadero St, San Francisco, CA 94115, USA; p4 Buck Institute for Research on Aging, 8001 Redwood Boulevard, Novato, CA 94945, USA; p5 University of Central Florida College of Medicine, Orlando, FL, USA; p6 Confluence Stat, Orlando, FL, USA

The letter by Carter et al. raises the important question of whether the design of our simulation model for investigating potential biases in the WISDOM trial (1) led to valid results.

We agree with Carter et al. that our model is a highly simplified representation of the complex breast cancer disease spectrum, and we are of course fully aware that the natural history of breast cancer involves the full spectrum of disease stages. We have in fact constructed a comprehensive simulation model representing all disease stages as well as impacts on costs and life-years gained from using different screening regimes. This model has been described in a manuscript that is currently under review. However, for this particular article, we were interested in a simple model to investigate as transparently as possible potential sources of bias in the primary analysis of WISDOM. We therefore chose to directly model the rate of stage IIB rather than modeling the entire natural history of breast cancer. Because rate of stage IIB or higher (ie, stage ≥ IIB) is the primary endpoint in WISDOM, it was sufficient to focus the simulation on this particular endpoint. Carter et al. write that with our simulation model, “one would expect similar proportions of stage IIB or higher disease in either study arm, leading one to conclude falsely the noninferiority of the personalized screening strategy.” This is in fact a deliberate design choice in our simulation model to investigate potential biases under the assumption that personalized screening is identical to annual screening for preventing stage IIB breast cancer, because that is the hypothesis we want to test in WISDOM. The use of this assumption in the simulations does not necessarily mean, of course, that personalized and annual screenings actually are identical. The fact that this is an open question is indeed why we perform the trial.
